# Perioperative use of point-of-care devices in cardiac surgery may improve bleeding and transfusion outcome

**DOI:** 10.1186/1749-8090-8-S1-O296

**Published:** 2013-09-11

**Authors:** R Habekovic, M Dubravec, D Zigrovic, S Gorupec-Kurtanjek, L Curak, V Berkovic, D Crnkovoic, S Beric, M Petricevic, B Biocina

**Affiliations:** 1 Department of Cardiac Surgery, University Hospital Center Zagreb, Zagreb, Croatia

## Background

Hemostatic disorder and massive bleeding are common complications after cardiac surgery procedures, particularly in procedures requiring prolonged cardiopulmonary bypass. Conventional laboratory coagulation tests failed to predict hemostatic disorder and consequent proclivity to excessive bleeding. On the other hand there is evergrowing proportion of patients who need antiplatelet therapy (APT) in perioperative setting. Platelet inhibitory response to antiplatelet therapy varies widely among individuals , thus reflecting bleeding tendency as well as risk for adverse ischemic events. Bedside suitable devises for assessment of platelet function as well as assessment of viscoelastic blood properties are desired. Herein, we present our institutional experience in hemostatic and antiplatelet therapy administration management.

## Methods

In 2008, we started with use of point-of-care devices at our Cardiac Surgery Department. We used following devices : Rotational thromboelastometry (ROTEM) , and Multiple electrode aggregometry (Multiplate).

ROTEM is an established method for assessment of viscoelastic blood clot properties in whole blood sample. ROTEM investigates the interaction of coagulation factors, their inhibitors, blood cells, specifically platelets, the role of fibrinogen during clotting and subsequent fibrinolysis.

Multiplate is based on the principle that blood platelets are nonthrombogenic in their resting state but expose receptors on their surface when they get activated, which allows them to attach onto vascular injuries and artificial surfaces. When platelets stick on the Multiplate sensor wires , they enhance the electrical resistance between them, which is continuously recorded, and expressed in arbitrary area under curve units.

## Results

At first, we found that both Multiplate and ROTEM parameters are predictives of postoperative bleeding extent. By Multiplate and ROTEM it was possible to stratify patients with respect to excessive bleeding risk. In addition to Multiplate identified patients who were resistant to APT, thus alowing for individual APT administration (type and dosage adjustment) management.

## Conslusion

In order to prevent excessive postoperative bleeding, hemostatic interventions with timely and targeted blood component therapy according to MEA and TEM results should be considered. Multiplate should be used for individual antiplatelet therapy administration management by determining adequate type and dosage of APT.

